# Protocol for the MoleMate™ UK Trial: a randomised controlled trial of the MoleMate system in the management of pigmented skin lesions in primary care [ISRCTN 79932379]

**DOI:** 10.1186/1471-2296-11-36

**Published:** 2010-05-11

**Authors:** Fiona M Walter, Helen C Morris, Elka Humphrys, Per N Hall, Ann Louise Kinmonth, A Toby Prevost, Edward CF Wilson, Nigel Burrows, Paul Norris, Margaret Johnson, Jon Emery

**Affiliations:** 1 General Practice & Primary Care Research Unit, Department of Public Health & Primary Care, University of Cambridge, Cambridge, UK; 2 School of Primary, Aboriginal and Rural Health Care, University of Western Australia, Crawley, Western Australia; 3 Addenbrooke's Hospital, Cambridge University Hospitals NHS Foundation Trust, Cambridge, UK; 4 King's College London, Department of Primary Care and Public Health Sciences, Capital House, London, UK; 5 Health Economics Group, Faculty of Health, University of East Anglia, Norwich, UK; 6 Lay member

## Abstract

**Background:**

Suspicious pigmented lesions are a common presenting problem in general practice consultations; while the majority are benign a small minority are melanomas. Differentiating melanomas from other pigmented lesions in primary care is challenging: currently, 95% of all lesions referred to a UK specialist are benign. The MoleMate system is a new diagnostic aid, incorporating a hand-held SIAscopy scanner with a primary care diagnostic algorithm. This trial tests the hypothesis that adding the MoleMate system to current best primary care practice will increase the proportion of appropriate referrals of suspicious pigmented lesions to secondary care compared with current best practice alone.

**Methods/design:**

The MoleMate UK Trial is a primary care based multi-centre randomised controlled trial, with randomisation at patient level using a validated block randomisation method for two age groups (45 years and under; 46 years and over). We aim to recruit adult patients seen in general practice with a pigmented skin lesion that cannot immediately be diagnosed as benign and the patient reassured. The trial has a 'two parallel groups' design, comparing 'best practice' with 'best practice' plus the MoleMate system in the intervention group. The primary outcome is the positive predictive value (PPV) of referral defined as the proportion of referred lesions seen by secondary care experts that are considered 'clinically significant' (i.e. biopsied or monitored). Secondary outcomes include: the sensitivity, specificity and negative predictive value (NPV) of the decision not to refer; clinical outcomes (melanoma thickness, 5 year melanoma incidence and mortality); clinician outcomes (Index of Suspicion, confidence, learning effects); patient outcomes (satisfaction, general and cancer-specific worry), and cost-utility.

**Discussion:**

The MoleMate UK Trial tests a new technology designed to improve the management of suspicious pigmented lesions in primary care. If effective, the MoleMate system could reduce the burden on skin cancer clinics of patients with benign pigmented skin lesions, and improve patient care in general practice.

## Background

### 1.1 Melanoma and health policy

Suspicious pigmented lesions (SPLs), or 'moles' are a common presenting problem in general practice consultations, and while the majority are benign, a small minority are malignant melanomas. Melanoma is a serious skin cancer, accounting for less than one in ten skin cancers, but responsible for 2% of all cancers and 1% of all cancer deaths in the UK, with around 9,500 new cases and 1,800 deaths a year (Cancer Research UK). Worldwide, the incidence of melanoma is increasing faster than any other cancer, with an approximate doubling of rates every 10-20 years in countries with white populations [1]. While the incidence in the UK is one of the highest in Europe, it is lower than the reported incidence from other countries such as Australia and New Zealand, which lead the world age-standardised rates at about 50 per 100,000 population per annum [2] (compared with 11.9 per 100,000 population in the UK (Cancer Research UK)). Furthermore, although the incidence of melanoma increases with age, a third of all cases occur in people aged less than fifty years and it is the second most common cancer in the 20-39 age group. The increasing incidence has been attributed to increases in ultra-violet exposure, both natural and artificial. Other risk factors include genetic predisposition reflected in the phenotypic features of multiple moles, fair complexion, sunburn-susceptible skin types, and family history. In the UK, melanomas are more common in women than in men, although the prognosis is poorer for men over 60 years who tend to present with thicker melanomas [3].

Early detection is critical in reducing mortality and morbidity from melanoma, as thin lesions (Breslow thickness less than 1 mm) have a five-year survival of over 95%, compared to less than 50% for thicker lesions (Breslow thickness more than 4 mm). There is evidence from the UK that expert consultation within 2 weeks of referral results in thinner tumours and improved overall survival [4]. The Department of Health's recent Cancer Reform strategy highlighted the issues of poor public awareness of cancer symptoms, diagnostic delays in primary care and late diagnosis across all cancers. In response, the National Awareness and Early Diagnosis Initiative was jointly launched by Cancer Research UK and the Department of Health in 2008 [5] with two of the seven key objectives being to develop interventions to promote early presentation and reduce diagnostic delays in primary care.

### 1.2 Primary care management of suspicious pigmented lesions

While some patients present late, the vast majority who present to their general practitioners (GPs) with concerns about moles or other pigmented skin lesions, will not be diagnosed with melanoma: even among men aged over 60 years, less than 1 in 33,000 moles are estimated to become malignant [6]. GPs need to be able to reassure those with benign lesions and rapidly refer those with suspicious lesions. This is a difficult challenge and, when compared with dermatologists, GPs can be highly sensitive but less specific for the diagnosis of melanoma [7,8]: consequently, the majority of lesions referred to skin cancer clinics in the UK prove to be benign [9]. Referral or diagnostic efficiency can be measured in terms of the ratio of benign to malignant lesions, or the proportion of referred lesions that are malignant. In 2003, the UK All Party Parliamentary Group on Skin reported that up to nineteen out of every twenty lesions referred to a dermatologist by a GP under the two-week cancer standard were benign. An evaluation of England's 2-week wait referrals showed that melanomas and squamous cell carcinomas accounted for 10 - 12%, with the remainder found to be benign lesions [10].

In an attempt to maximise sensitivity and specificity of melanoma diagnosis by GPs and their referral efficiency, a variety of educational, checklist and technical approaches have been developed. There is equivocal evidence about the usefulness of training courses: while randomised controlled trial evidence from the US suggests some benefit from face-to-face [11] and internet [12] training, Australian studies have shown that face-to-face training of GPs in melanoma diagnosis has no significant effect on their performance [7]. The ABCD [13] and seven-point [14] checklists have been widely evaluated and revised, but each has significant drawbacks in helping GPs distinguish between benign pigmented lesions and melanomas [15]. Technical approaches include the use of dermoscopy, laser microscopy, ultrasonography and magnetic resonance imaging [16]. A recent trial of dermoscopy and digital monitoring in Australian general practice found that the combination of these techniques can increase the sensitivity of GPs for the diagnosis of melanoma, and significantly reduce the proportion of benign lesions excised [17]. However, learning these techniques took considerable time and was completed by only 62% of trial GPs. Therefore, other approaches that are easier to learn are still required to improve GPs' diagnostic performance and support the assessment of pigmented skin lesions in primary care.

### 1.3 SIAscopy and the MoleMate system

SIAscopy is an innovative technology that uses a non-invasive multispectral scanning technique to produce images of the light-absorbing chromaphores haemoglobin, melanin and collagen, in the epidermis and papillary dermis. Patterns within the SIAscans of pigmented skin lesions have been shown to indicate histopathological and pathophysiological features consistent with melanoma, and, in combinations, these features were highly sensitive (83%) and specific (80%) for melanoma in the experimental setting [18]. The Moncrieff Scoring System was developed and, in a population referred to a skin cancer clinic, was shown to have 94.4% sensitivity and a 3.7% false positive rate for melanoma [19]. In the primary care setting the Moncrieff Scoring System was less sensitive for the diagnosis of 'suspicious' lesions (54.2%) and melanoma (66.7%), and a new Primary Care Scoring Algorithm (PCSA) was developed to account for the higher prevalence of seborrhoeic keratoses and haemangiomas seen in primary care. This had a similar sensitivity for the diagnosis of 'suspicious' (50.0%) but was more specific (84.2%). Based on simulation modelling, the PCSA also demonstrated high sensitivity and specificity for the diagnosis of melanoma (94.0% and 83.5% respectively) [20,21].

Subsequently the PCSA was incorporated into the MoleMate system. This randomised controlled trial (RCT), set in UK general practice, aims to test the hypothesis that use of the MoleMate system will improve the management of suspicious pigmented lesions in primary care compared with current best practice, and that this will be reflected in the appropriateness of referrals to skin cancer clinics.

## Methods/designs

### 2.1 Definitions

For the purpose of this trial, the definition of 'suspicious pigmented lesion' is any lesion presented by a patient, or opportunistically seen by a clinician in general practice, which cannot immediately be diagnosed as benign and the patient reassured.

The definition of 'appropriateness of referral' is whether the referred lesion is deemed 'clinically significant' by a dermatologist in a skin cancer clinic and is therefore either biopsied or monitored. Those lesions seen by a dermatologist in a skin cancer clinic which result in reassurance and discharge from the clinic without biopsy or monitoring are deemed 'clinically insignificant'.

### 2.2 Sponsorship and ethical approval

The trial is jointly sponsored by the University of Cambridge and Cambridgeshire Primary Care Trust (now renamed NHS Cambridgeshire). Cambridgeshire 2 Research Ethics Committee has reviewed and approved the protocol (07/H0308/167).

### 2.3 Trial design and randomisation

**Figure 1:**
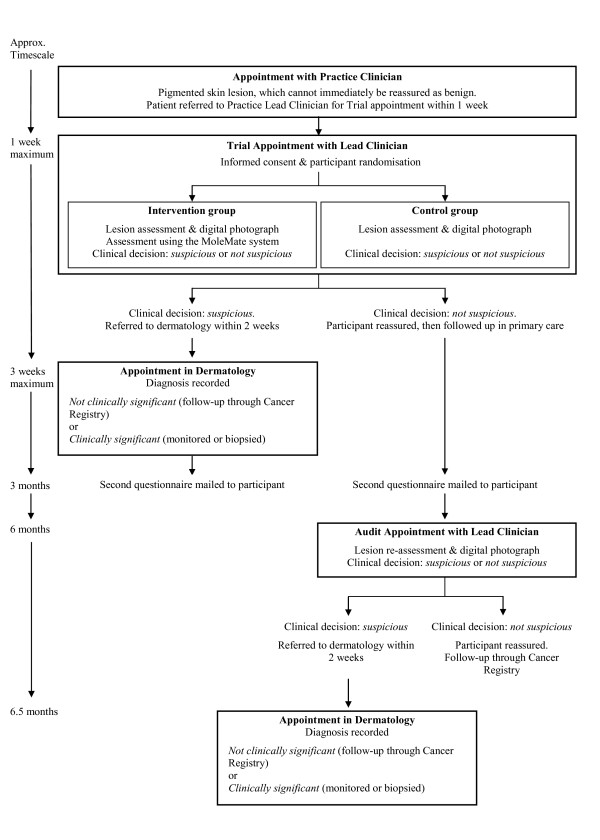
**Trial flowchart**.

This randomised controlled trial comprises 2 stages: a pilot phase and the main trial. The pilot phase was conducted in 3 general practices over a 4-month period; during this time the study procedures were refined and projected recruitment rates confirmed. The study design and flow of participants through the trial are shown in Figure 1. Once eligible patients have been internally referred within the general practice to one of the trial Lead Clinicians and recruited into the trial, participants are randomised to either the comparison ('best practice') group or the intervention (MoleMate system) group. Participants are randomised using a block randomisation method with sets of numbered, sealed envelopes prepared by the trial statistician (ATP), with the order of the sequences used being verified afterwards. Randomisation is stratified by age (45 years and under; 46 years and over) and by Lead Clinician.

### 2.4 Setting

Fifteen general practices are participating in the trial: thirteen in Cambridgeshire, one in West Essex and one in Suffolk. Practices already using a MoleMate system have been excluded. Two Lead Clinicians per practice have undergone training in the use and interpretation of the MoleMate system, and in all study procedures. GPs with known dermatological expertise eg GPs with a special interest (GPSI), Hospital Practitioners and Clinical Assistants in Dermatology, were excluded from being Lead Clinicians.

### 2.5 Patients

Patients are eligible for the trial if aged 18 years or over, able to give informed consent, and have a pigmented lesion (either presented by the patient or noticed by a health care professional) which is not immediately diagnosed as benign and the patient reassured. These patients are given an appointment with a Lead Clinician within one week and provided with information about the MoleMate Trial prior to formal consent and recruitment by the Lead Clinician.

### 2.6 Study procedures

At the trial consultation, the Lead Clinician consents and randomises the patient into the 'comparison' or 'intervention' group.

#### Comparison group

Participants in the comparison group have their lesion assessed in the usual 'best practice' manner using the clinical history, naked eye examination and the seven-point checklist [14].

#### Intervention group

Prior to commencing patient recruitment, Lead Clinicians complete a MoleMate Training CD-rom which teaches users to identify relevant SIAscopic features of various pigmented skin lesions. The Training CD-rom takes up to 2 hours to complete and has been shown to significantly improve GPs' ability to interpret SIAscopic images [22]. Participants in the intervention arm have their lesion/s assessed according to the same 'best practice' principles in the comparison arm. Lead Clinicians then use the MoleMate system to support their assessment and management of the lesion.

For all lesions an Index of Suspicion scale is completed, and the clinical diagnosis and intended action recorded (suspicious and refer via the two-week cancer route, or benign and reassure). A digital photograph is taken of every lesion for expert review.

### 2.7 Reference standards

Although the true 'gold standard' would be histology on all lesions, this would not be practical or ethically acceptable for every participant. In clinical practice, an expert opinion is considered the practical 'standard' of whether or not a pigmented lesion is 'clinically significant' in requiring monitoring or biopsy.

#### 2.7.1 Referred lesions

All lesions considered 'suspicious' by a Lead Clinician from both trial arms are referred to secondary care (Addenbrooke's Hospital) via the normal two-week skin cancer care pathway, where one of three dermatologists (Experts A, B and C) provide an opinion as to whether or not the pigmented lesion is 'clinically significant'.

#### 2.7.2 Non-referred lesions

All lesions considered 'not suspicious' by a Lead Clinician, and therefore not referred, are assessed three months later by two other skin cancer experts (Experts D and E). The expert assessment includes review of the seven-point checklist, the digital photograph and, for intervention group participants, the MoleMate images to make the final reference standard diagnosis of 'suspicious' or 'not suspicious'.

### 2.8 Safety procedures

All non-referred participants with a lesion considered 'suspicious' by Experts D and E are invited to an early review by the Lead Clinician to re-assess the lesion and thus ensure safety in the study. All other participants with 'not suspicious' lesions, as determined by Experts D and E, are invited to attend an Audit Appointment with the Lead Clinician six months after their first consultation. At this consultation the lesion is re-examined and the seven-point checklist and digital photograph repeated. Experts D and E compare these new data with the original trial data to ensure no potentially suspicious lesions are 'missed' within the trial. This safety-net procedure was introduced in response to concerns raised by the Research Ethics Committee.

### 2.9 Measurement

Table 1 summarises the content and timing of all the clinical and psychological measures.

**Table 1:** Trial measures and timing.

		Time after 1^st ^Lead Clinician (LC) consultation				
	
		All participants			Non-referred participants	Referred participants
	
		1^st ^LC consultation	up to 1 week	3 months	6 months	within 2 weeks
**Clinical assessment of lesion(s)**		√			√	√
					
**Psychological measures (questionnaire)**						
Anxiety: short form STAI^†^			√	√		
Cancer worry: modified cancer worry scale			√	√		
Satisfaction with LC consultation^¥^			√			

^† ^Spielberger State-Trait Anxiety Inventory

^¥ ^Items from Europep instrument relating to the dimensions of care

#### 2.9.1 Primary outcome

The primary outcome is the positive predictive value (PPV) of the primary care decision to refer. This is defined as the proportion of referred lesions that are considered 'clinically significant' (biopsy or monitor) by secondary care experts, and is therefore the proportion of all referred lesions that are deemed 'clinically significant'. This is a measure of the appropriateness of a referral and, by implication, the diagnostic accuracy of the GP with or without the aid of the MoleMate system.

#### 2.9.2 Secondary outcomes

##### Related to the primary outcome

- Volume of referrals: there is a possibility that the proportion of appropriate referrals will be the same in the Intervention and Comparison groups despite differences between these groups in the number of total referrals (denominator) and the number of clinically significant lesions (numerator). The primary outcome measure will be unpacked to examine the total number of referrals (i.e. volume) and the number of clinically significant lesions in each group;

- Sensitivity, specificity and negative predictive value (NPV) of the decision not to refer using data from all lesions in the trial.

##### Clinical outcomes

- Thickness of melanomas biopsied in secondary care clinics;

- 5 year melanoma incidence and mortality among trial participants collected through the Eastern Cancer Registration and Information Centre (ECRIC).

##### Clinician outcomes

- Accuracy of Index of Suspicion scale;

- Confidence and attitudes of Lead Clinicians towards the MoleMate intervention will be assessed 2 weeks after trial set-up and at trial completion using a modified measure based on the Theory of Planned Behaviour [23];

- Learning effects: it is possible that the MoleMate training programme, and using the MoleMate system in practice, may improve the Lead Clinicians' accuracy to assess pigmented lesions clinically and thereby affect management of lesions in both groups. We will therefore examine potential contamination effects by comparing the appropriateness and volume of referral between groups for the first ten MoleMate consultations of each Lead Clinician's data collection when contamination would be minimal ('naive period'), with the remaining consultations ('potentially contaminated period').

##### Patient outcomes

Participants will complete the following psychological measures within 1 week and at 3 months after their consultation with the Lead Clinician:

- General and cancer-specific worry: Anxiety using the Spielberger State-Trait Anxiety Inventory, STAI [24] and a modified cancer worry scale [25];

- Satisfaction with the trial consultation using dimensions of care items from Europep [26].

##### Resource use and costs

The cost of the MoleMate system to the NHS will be assessed using the following data: costs of the MoleMate system (purchase, maintenance and technical support, staff training), costs of primary care activity (initial and diagnostic GP visits), and costs of secondary care activity (referrals, excision).

### 2.10 Sample size

Our pilot study confirmed projected recruitment rates (6-8 participants per practice per month) and referral rates (approximately 2 referrals per practice per month). Based on these, to detect an increased biopsy rate of 10% amongst those referred of 22% ('intervention' group) versus 12% ('comparison' group), an odds ratio of 2.0, with 80% power at the 5% significance level, would require 442 analysed in the primary outcome sample of referred lesions. With the involvement of 15 practices, this would require 30 referred lesions per practice. Assuming a 25% referral rate this could be achieved by randomising 120 patients per practice (1800 total) with an assumed recruitment rate of 90 per year over a 16-month period.

A blinded interim analysis, presented at the Trial Steering Committee meeting half-way through the study (September 2009) after 747 participants had been randomised, established a lesion referral rate of 28% (254/913), a mean cluster size amongst the referred sample of 1.06 (254 lesions from 239 participants), and that 55% of the referred lesions were assessed by the dermatology experts to be 'clinically significant' in the combined arm data. In this context of a higher rate of biopsy or monitoring, the clinically worthwhile difference was altered to a 15% difference (an odds ratio of 1.8) from 45% to 60%. A target total of 400 referred lesions from approximately 380 patients would provide 80% power to detect this difference at the 5% level of significance, and would require an estimated 1450 study lesions from 1150 randomised participants.

### 2.11 Analysis

#### Primary outcome (PPV of referral)

Analysis will be by intention-to-treat using the referred population for the primary outcome (i.e. all lesions referred to secondary care). The proportion of all referred lesions that are deemed 'clinically significant' will be compared between randomised groups using Donner's test for clustered proportions via a linear mixed effects model with patient as a random term. A secondary analysis comparing the proportions (chi-squared test) without allowance for clustering will enable an assessment as to whether clustering makes any difference to the results. If there is no material difference the simple analysis without need of accounting for clustering will be reported.

#### Secondary outcomes

##### 1. Related to the primary outcome

*1.1 Volume effects*

The volume of referrals will be measured by the percentage of randomised participants over the study period who are referred by Lead Clinicians to secondary care, and compared between randomised groups as a difference in proportions with 95% confidence interval for the difference, and using the chi-squared test.

*1.2 Sensitivity, specificity and negative predictive value of the decision not to refer*

Sensitivity, specificity and other study proportions of lesions will be analysed using the same approach as for the primary outcome measure. These data will be used to compare the PPV of a referral (the primary outcome), and the NPV (correct management of lesions not referred) which will provide important data around lesions which are incorrectly identified as not suspicious in general practice. These data can contribute to further development of the diagnostic algorithm embedded in the MoleMate system.

##### 2. Clinical outcomes

Histological diagnosis of melanoma will be summarised as a rate in each randomised group, with the denominator being the participants in each randomised group: exact methods (95% confidence interval and Fishers exact test) will be used to estimate the difference in rates of melanoma. Melanoma thickness (Breslow data) and other histological diagnoses will be summarised using the same approach.

From five-year Cancer Registry follow-up for all participants (incidence and mortality) survival analysis methods will be used, including the log rank test, Kaplan Meier survival plots, and Cox regression to estimate intervention effects with 95% confidence intervals and associated p-value (involving the full participant sample in each randomised group (referred plus non-referred) to assess overall effect of the introduction of the MoleMate system compared to current best practice).

##### 3. Clinician outcomes

Continuous outcomes at the level of clinician such as confidence and attitudes, and change in these, towards the MoleMate system will be summarised using a mean score and 95% confidence interval and reporting the proportion of clinicians above a threshold of adequate confidence.

Assessment for learning effects will be undertaken using randomised comparisons of differences in proportions across 'naive' and 'potentially contaminated' periods: if there is an improvement in performance in the comparison arm and the intervention effect size becomes smaller through Lead Clinicians' sequence of lesions assessed, and this is a systematic effect established across the study clinicians, this would be evidence of either a learning effect or more general contamination.

The diagnostic performance of the seven-point checklist and the Index of Suspicion scale will be assessed and compared between the intervention and comparison groups.

##### 4. Patient outcomes

Continuous secondary outcomes at the participant level, such as anxiety, cancer worry, and satisfaction with the consultation will be compared using a t-test, assuming no clustering effects are identified. Analyses will be unadjusted cross-sectional comparisons between groups. Change over time will also be summarised, reporting means at baseline and mean changes between time-points.

##### 5. Economic assessment

The economic evaluation will be a cost-utility analysis conducted from the perspective of the UK National Health Service, assessing the incremental quality adjusted life years (QALYs) gained and incremental cost to the NHS from the MoleMate system versus current best practice. The incremental cost-effectiveness ratio will be calculated, and appropriate analysis of uncertainty will be undertaken. Drawing on the trial and other epidemiological and cost data from the literature, the analysis will be conducted over a 10 year time horizon, and based on a decision tree, defined at the point of analysis.

### 2.12 Trial organisation and management

A Trial Steering Committee, consisting of investigators, research team members, expert dermatologists and plastic surgeons, a statistician, a health economist, Biocompatibles plc (previously Astron Clinica) staff, two lay members and an independent chair, has been formed and will meet annually for the duration of the trial.

## Discussion

This trial is designed to estimate the effect of the MoleMate intervention, compared with current best practice, on the management of suspicious pigmented lesions in general practice, as reflected in the appropriateness (PPV) of referrals to a skin cancer clinic. As such, we are considering the MoleMate system as a diagnostic tool and testing its effectiveness in the hands of primary care clinicians. There has been increasing interest in clinical decision support (CDS) aids in recent years, and they range from electronic texts, drug information and practice guideline databases to rules-based guidance systems that direct clinicians about exactly what to do for specific clinical problems. The MoleMate system is a good example of a CDS aid, with the incorporation of an algorithm specifically developed for use in primary care, with a new imaging technology already increasingly used by dermatologists. The MoleMate system has been developed to extend the utility of SIAscopy from differentiating melanoma from other pigmented lesions in the secondary care setting, to differentiating 'suspicious lesions' from 'non suspicious lesions' in the primary care setting.

The trial seeks to address a common challenge for primary care health professionals- how to recognise and appropriately manage the rarely-presenting but serious conditions from commonly-presenting benign conditions. Differentiating melanomas from other pigmented lesions in primary care is particularly challenging, even among men aged over 60 years more than 99.7% are estimated to remain benign [6], and 95% of all lesions currently referred to a specialist prove to be benign [10]. The development and rigorous evaluation of tools such as the MoleMate system to assist clinical decision making in primary care is one approach to maximising referral efficiency, and as such meets one objective of the National Awareness and Early Diagnosis Initiative [5].

The measures and interventions for the MoleMate UK Trial have been developed using the approach defined by the MRC Framework for development of complex interventions for evaluation in randomised trials [27,28] to ensure rigour, transparency and reproducibility. The four-month pilot phase confirmed the proposed recruitment and referral rates and the feasibility of participant randomisation. It demonstrated the feasibility of tracking referred participants in secondary care, and established mechanisms for reference standard diagnosis for all lesions recruited into the trial. During the pilot phase semi-structured interviews and questionnaires confirmed that both participants and lead clinicians found all aspects of the trial process, including randomisation, were acceptable and did not appear to cause distress. However, we await data from the main trial on the psychological impact of trial participation and acceptability of the MoleMate system.

This trial tests a novel service model of internal referral in general practice to Lead Clinicians who do not have dermatological training. We are conducting an economic evaluation of the use of the MoleMate system within this service model to establish its cost-utility compared with current best practice.

Our primary outcome measure is based on the clinical significance of a lesion, as determined by the actual management (biopsy or monitor) by a skin cancer specialist. This is a meaningful outcome in terms of health service delivery and the need to improve referral efficiency. While ideally we would have detection of melanoma as the primary outcome, this would require a much larger trial to have sufficient statistical power. We will capture data on melanoma incidence and mortality in all participants over 5 years. This will provide important data on the false negatives; lesions incorrectly diagnosed as benign.

## Conclusions

In conclusion, this is the first randomised controlled trial in general practice of a decision aid using SIAscopy for the assessment of pigmented skin lesions. Preliminary data from diagnostic validation studies in the UK and Australia suggest the MoleMate system is reasonably sensitive and specific but only an RCT can determine its effect on clinical management in the UK setting. If the trial demonstrates that using the MoleMate system improves diagnostic efficiency, it will add to the range of approaches to support assessment of pigmented skin lesions in UK primary care. If the trial provides evidence of cost-utility it will inform new service models to reduce referrals to secondary care of benign lesions and improve the early diagnosis of melanoma.

## Competing interests

The authors declare that they have no competing interests.

## Authors' contributions

The writing group for the MoleMate UK Trial Protocol comprised: Fiona M Walter (FW), Jon Emery (JE), Per Hall (PH), Ann Louise Kinmonth (ALK) and Toby Prevost (ATP) (all principal investigators), with the MoleMate UK Trial team members: Helen Morris (HM, Trial Coordinator), Elka Humphrys (EH, Research Assistant), Nigel Burrows (NB) and Paul Norris (PN, consultant dermatologists); Ed Wilson (EW, trial health economist), and Margaret Johnson (lay member).

FW, JE and PH conceptualised the research. FW, JE, PH, ALK, ATP and HM conceived the development of study design. FW and HM, assisted by JE, oversaw the running of the trial and drafted the protocol paper. EW drafted the economic evaluation component. EH contributed to the finalisation of the study design following the pilot phase. All authors read and approved the final manuscript.

## Pre-publication history

The pre-publication history for this paper can be accessed here:

http://www.biomedcentral.com/1471-2296/11/36/prepub

## References

[B1] Lens MB Dawes M Global perspectives of contemporary epidemiological trends of cutaneous malignant melanoma British Journal of Dermatology 2004 150 179 185 10.1111/j.1365-2133.2004.05708.x 14996086

[B2] Parkin DM Bray FI Devesa SS Cancer burden in the year 2000. The global picture Eur J Cancer 2001 37 Suppl 8 S4 66 10.1016/S0959-8049(01)00267-2 11602373

[B3] MacKie RM Bray C Vestey J Doherty V Evans A Thomson D Nicolson M Melanoma incidence and mortality in Scotland 1979-2003 Br J Cancer 2007 96 1772 1777 10.1038/sj.bjc.6603801 17533392 PMC2359933

[B4] Pacifico MD Pearl RA Grover R The UK Government two-week rule and its impact on melanoma prognosis: an evidence-based study Ann R Coll Surg Engl 2007 89 609 615 10.1308/003588407X205459 18201477 PMC2121231

[B5] CRUK The National Awareness and Early Diagnosis Inititative (NAEDI) 2008

[B6] Tsao H Bevona C Goggins W Quinn T The transformation rate of moles (melanocytic nevi) into cutaneous melanoma: a population-based estimate Arch Dermatol 2003 139 282 288 10.1001/archderm.139.3.282 12622618

[B7] Burton RC Howe C Adamson L Reid AL Hersey P Watson A Watt G Relic J Holt D Thursfield V Clarke P Armstrong BK General practitioner screening for melanoma: sensitivity, specificity, and effect of training J Med Screen 1998 5 156 161 9795877 10.1136/jms.5.3.156

[B8] Chen SC Pennie ML Kolm P Warshaw EM Weisberg EL Brown KM Ming ME Weintraub WS Diagnosing and managing cutaneous pigmented lesions: primary care physicians versus dermatologists J Gen Intern Med 2006 21 678 682 10.1111/j.1525-1497.2006.00462.x 16808765 PMC1924688

[B9] Morrison A O'Loughlin S Powell FC Suspected skin malignancy: a comparison of diagnoses of family practitioners and dermatologists in 493 patients Int J Dermatol 2001 40 104 107 10.1046/j.1365-4362.2001.01159.x 11328390

[B10] Cox NH Evaluation of the U.K. 2-week referral rule for skin cancer Br J Dermatol 2004 150 291 298 10.1111/j.1365-2133.2004.05793.x 14996100

[B11] Gerbert B Bronstone A Wolff M Maurer T Berger T Pantilat S McPhee SJ Improving primary care residents' proficiency in the diagnosis of skin cancer J Gen Intern Med 1998 13 91 97 10.1046/j.1525-1497.1998.00024.x 9502368 PMC1496907

[B12] Gerbert B Bronstone A Maurer T Berger T McPhee SJ Caspers N The effectiveness of an Internet-based tutorial in improving primary care physicians' skin cancer triage skills J Cancer Educ 2002 17 7 11 12000111 10.1080/08858190209528784

[B13] Friedman RJ Rigel D Kopf AW Early detection of malignant melanoma: the role of physician examination and self-examination of the skin CA Cancer J Clin 1985 35 130 151 10.3322/canjclin.35.3.130 3921200

[B14] MacKie RM Doherty VR Seven-point checklist for melanoma Clin Exp Dermatol 1991 16 151 153 10.1111/j.1365-2230.1991.tb00329.x 1867692

[B15] Liu W Hill D Gibbs AF Tempany M Howe C Borland R Morand M Kelly JW What features do patients notice that help to distinguish between benign pigmented lesions and melanomas?: the ABCD(E) rule versus the seven-point checklist Melanoma Res 2005 15 549 554 10.1097/00008390-200512000-00011 16314742

[B16] Marghoob AA Swindle LD Moricz CZ Sanchez Negron FA Slue B Halpern AC Kopf AW Instruments and new technologies for the in vivo diagnosis of melanoma J Am Acad Dermatol 2003 49 777 797 10.1016/S0190-9622(03)02470-8 14576657

[B17] Menzies SW Emery J Staples M Davies S McAvoy B Fletcher J Shahid KR Reid G Avramidis M Ward AM Burton RC Elwood JM Impact of dermoscopy and short-term sequential digital dermoscopy imaging for the management of pigmented lesions in primary care: a sequential intervention trial Br J Dermatol 2009 161 1270 1277 10.1111/j.1365-2133.2009.09374.x 19747359

[B18] Moncrieff M Cotton S Claridge E Hall P Spectrophotometric intracutaneous analysis: a new technique for imaging pigmented skin lesions Br J Dermatol 2002 146 448 457 10.1046/j.1365-2133.2002.04569.x 11952545

[B19] Govindan K Smith J Knowles L Harvey A Townsend P Kenealy J Assessment of nurse-led screening of pigmented lesions using SIAscope J Plast Reconstr Aesthet Surg 2007 60 639 645 10.1016/j.bjps.2006.10.003 17485052

[B20] Hunter JE Triaging suspicious pigmented skin lesions in primary care using the SIAscope 2008 MD, University of Cambridge

[B21] Emery J Hunter JE Watson A Hall PN Moncrieff M Walter FM Accuracy of SIAscopy for pigmented skin lesions encountered in primary care: development and validation of a new diagnostic algorithm BMC Dermatology in press 10.1186/1471-5945-10-9 PMC2954906 20868511

[B22] Wood A Morris H Emery J Hall PN Cotton S Prevost AT Walter FM Evaluation of the MoleMate training program for assessment of suspicious pigmented lesions in primary care Inform Prim Care 2008 16 41 50 18534076 10.14236/jhi.v16i1.673

[B23] Emery J Morris H Goodchild R Fanshawe T Prevost AT Bobrow M Kinmonth AL The GRAIDS Trial: a cluster randomised controlled trial of computer decision support for the management of familial cancer risk in primary care Br J Cancer 2007 97 486 493 10.1038/sj.bjc.6603897 17700548 PMC2360348

[B24] Marteau TM Bekker H The development of a six-item short-form of the state scale of the Spielberger State-Trait Anxiety Inventory (STAI) Br J Clin Psychol 1992 31 Pt 3 301 306 1393159 10.1111/j.2044-8260.1992.tb00997.x

[B25] Lerman C Schwartz MD Lin TH Hughes C Narod S Lynch HT The influence of psychological distress on use of genetic testing for cancer risk Journal of Consulting 1997 65 414 420 10.1037/0022-006X.65.3.414 9170764

[B26] Grol R Wensing M Mainz J Jung HP Ferreira P Hearnshaw H Hjortdahl P Olesen F Reis S Ribacke M Szecsenyi J Patients in Europe evaluate general practice care: an international comparison Br J Gen Pract 2000 50 882 887 11141874 PMC1313852

[B27] Campbell NC Murray E Darbyshire J Emery J Farmer A Griffiths F Guthrie B Lester H Wilson P Kinmonth AL Designing and evaluating complex interventions to improve health care BMJ 2007 334 455 459 10.1136/bmj.39108.379965.BE 17332585 PMC1808182

[B28] Anderson R New MRC guidance on evaluating complex interventions BMJ 2008 337 a1937 10.1136/bmj.a1937 18945728

